# The chemoreceptor genes of the waterflea *Daphnia pulex*: many Grs but no Ors

**DOI:** 10.1186/1471-2148-9-79

**Published:** 2009-04-21

**Authors:** D Carolina Peñalva-Arana, Michael Lynch, Hugh M Robertson

**Affiliations:** 1 Department of Biology, Indiana University, Bloomington, IN 47405, USA; 2 Department of Entomology, University of Illinois at Urbana-Champaign, Urbana, IL 61801, USA

## Abstract

**Background:**

Chemoreception is vitally important for all animals, yet little is known about the genetics of chemoreception in aquatic organisms. The keystone species *Daphnia pulex*, a well known crustacean, is the first aquatic invertebrate to have its genome sequenced. This has allowed us the initial investigation of chemoreceptor genes in an aquatic invertebrate, and to begin the study of chemoreceptor evolution across the arthropod phylum.

**Results:**

We describe 58 Grs (gustatory receptors), belonging to the insect chemoreceptor superfamily, which were identified bioinformatically in the draft genome of the crustacean waterflea *Daphnia pulex*. No genes encoding proteins similar to the insect odorant receptors (Ors) were identified. These 58 Grs form 3 distinctive subfamilies of 37, 12, and 5 genes, as well as a highly divergent singleton (Gr58). In addition, Grs55–57 share distinctive amino acid motifs and cluster with the sugar receptors of insects, and may illuminate the origin of this distinctive subfamily. ESTs, tiling array, and PCR amplification results support 34 predicted gene models, and preliminary expression data comparing the sexes indicates potential female-biased expression for some genes.

**Conclusion:**

This repertoire of 58 chemoreceptors presumably mediates the many chemoperception abilities of waterfleas. While it is always possible that the entire Or gene lineage was lost at some point in the history of *Daphnia pulex*, we think it more likely that the insect Or lineage is indeed a relatively recently expanded gene lineage concomitant with the evolution of terrestriality in the insects or their hexapod ancestors.

## Background

The ability of *Daphnia *to detect chemical cues released by prey or predator have been glimpsed through studies on feeding behavior and predator avoidance [1,2]. Daphnids reject food particles, adjust feeding currents according to food availability, quality and surrounding chemical cues, and appear to swim and remain in areas where food is abundant [3-6]. However, their presence in food abundant areas can be altered by the presence of predators, and their vertical distribution is often associated with the presence or absence of predators [6,7].

Kairomones from predators, fish or invertebrate, affect *Daphnia's *swimming patterns, dial vertical migration, and even affect morphology [2,8-11]. Species of *Daphnia *can develop neck teeth, thicker carapaces, and/or long head spines to reduce their vulnerability to predation [12,13]. Predator chemical signals, both fish and invertebrate kairomones, share some similarities. For example, in the case of *Leucaspius delineates *and *Chaoborus americanus*, these kairomones are made up of more than one active component with low-molecular weight and are organic water-soluble molecules with intermediate polarity, that have no primary amines and require hydroxyl groups for activity; they are also heat stable molecules that can be partially destroyed by acid and base digestions [14,15]. When the kairomones of different fish species were compare there was a striking resemblance between both groups, indicating that the signals are very similar if not identical and are found free in solution and not bound to edible particles [15]. However progress is still slow in the identification of the molecular nature of kairomones, and we are yet to isolate any individual chemical that can invoke a robust chemical mediated behavior in aquatic invertebrates [16].

The cues involved in *Daphnia *mating are not well understood. Unlike copepod males that can trace a female signal in the water column [17], *Daphnia *males must come into contact with potential mates [18,19]. This inspection is quick and although the cues needed to tell sexes and species apart could be mechanical; it is also possible that a female pheromone is present on the sexual female's carapace, allowing males to quickly identify a mate. The possibility that a chemical cue is involved in mating is hinted at by the fact that males press their antennules against a potential mate, and these antennules are structurally identical to known chemosensors found on other crustaceans and the first antennae of terrestrial insects (also known as chemosensors) [20,21].

In insects a chemoreceptor superfamily of seven-transmembrane domain proteins (TM7) provides the molecular basis for the specificity and sensitivity of both smell and taste (recently reviewed by [22-25]). The superfamily consists of the gustatory receptor (Gr) family [26-28], which contains most of the protein diversity of the superfamily [29], and the odorant receptor (Or) family [30-32], which is a single highly expanded lineage [29]. The Or superfamily has now been described in a variety of insects. These include both endopterygote relatives of the *Drosophila melanogaster *fruitflies in which they were first discovered, for example, the other 11 *Drosophila *species with genome sequences [33-36], as well as the mosquitoes *Anopheles gambiae *[37] and *Aedes aegypti *[38,39], the silkworm moth *Bombyx mori *[40,41], the red flour beetle *Tribolium castaneum *[42,43], and the honey bee *Apis mellifera *[44]. While this chemoreceptor superfamily is clearly very old with distant relatives of the Grs identified in the *Caenorhabditis *nematodes, Robertson et al. (2003) suggested that the Ors might be a relatively recent expansion of dedicated odorant receptors from a particular Gr lineage concomitant with the evolution of terrestriality in insects from a crustacean ancestor. The availability of a draft genome sequence for the waterflea *Daphnia pulex *[45], a representative of the freshwater branchiopod crustaceans thought by some to be the sister group to the terrestrial insects (e.g. [46]), allows a first test of this proposal.

Here we describe the chemoreceptor superfamily revealed by the draft genome sequence for *D. pulex*, finding six lineages of Grs, including one expanded to 37 genes, for a total of 58 genes. These presumably mediate the many "taste" functions in this freshwater crustacean. Consistent with the prediction of Robertson et al. (2003), we find no evidence of Ors. This includes the basal and highly conserved ortholog of the unusual DmOr83b protein implicated in partnering with each of the specific Ors in individual olfactory sensory neurons [47-52]. While it is always possible that this entire Or gene lineage was lost at some point in the history of *Daphnia pulex*, we think it more likely that the insect Or lineage is indeed a relatively recently expanded gene lineage concomitant with the evolution of terrestriality in the insects or their hexapod ancestors.

## Results

### Absence of Ors

Extensive BLASTP searches of the predicted proteins encoded by the v1.0, NCBI GNOMON, and merged v1.1 gene builds provided by the JGI at DOE, as well as TBLASTN searches of the September 2006 draft genome sequence using representative Grs and Ors from all available insects as queries revealed only multiple lineages of Grs. In particular, no homolog of the otherwise highly conserved DmOr83b protein, which has orthologs in all available insect genomes, was identified. It is always possible that a particular gene might be in a region of a genome that cloned poorly in the genomic libraries employed in a genome project, and hence was sequenced too thinly to be assembled. We therefore also searched all 2,724,768 raw traces deposited in the Trace Archive at GenBank using the TBLASTN algorithm for any reads with sequence similarity to all available DmOr83b orthologs from insects, and found none. Similar searches with representative insect Ors similarly revealed no convincing matches. We conclude that the *D. pulex *genome does not encode a homolog of the DmOr83b protein or any other insect Or homologs and that the entire insect Or gene family is absent from this crustacean genome.

### A diversity of Grs

**Figure 1:**
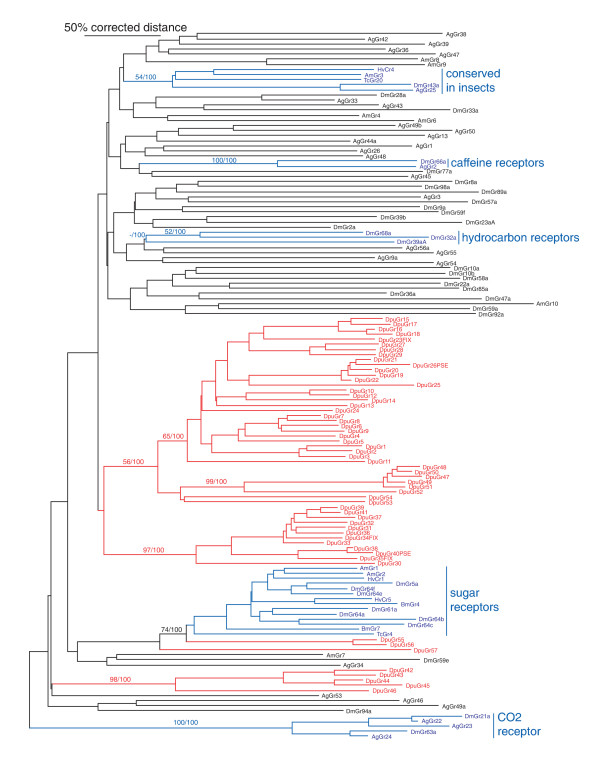
**Phylogenetic relationships of the 58 *Daphnia pulex *Grs to each other and a representative set of insect Grs**. This is a corrected distance tree, with the highly conserved CO2 receptor lineage designated as the outgroup to root the tree. Bootstrap values from 10,000 replications of uncorrected distance analysis are shown on major branches, followed by Bayesian posterior probabilities. DpuGr (*D. pulex*) lineages are highlighted in red. Major groups of insect Grs whose ligands are known or which are mentioned in the text are highlighted in blue (Ag – *Anopheles gambiae*, Am – *Apis mellifera*, Bm-*Bombyx mori*, Hv- *Heliothis virescens, and *Tc- *Tribolium castaneum*).

We identified fifty eight genes encoding proteins belonging to the Gr family (Table 1 and Figure 1). About half of these genes are found in tandem arrays across 21 scaffolds in the sequenced genome (Table 1). While genes within tandem arrays are usually phylogenetic close to each other in the tree, there has been considerable gene movement within the genome. For example, although Grs1–9 cluster together in the tree, they are in three tandem arrays spaced across 2 Mbp on scaffold 4. Grs47–52 form a phylogenetic cluster, and most are in a tandem array on scaffold 2, but Gr47 is on scaffold 58.

**Table 1:** *Daphnia pulex *gustatory receptor (Gr) gene model support.

**DpuGr**	**Location**	**JGI V1.1 ** **gene model**	**Protein ID**	**New Protein ** **ID**	**Comments**
1	scaffold_4:272236-273762	fgenesh1_pg.C_scaffold_4000034	346811	NA	Same

2	scaffold_4:278009-279502	NCBI_GNO_0400033	311261	346819	truncated 1st exon

3	scaffold_4:279988-281469	NCBI_GNO_0400034	311262	346813	missing final exon

4	scaffold_4:341660-343135	fgenesh1_pg.C_scaffold_4000053	95937	346911	truncated 1st exon

5	scaffold_4:339828-341493	PASA_GEN_0400197	305579	NA	Same

6	scaffold_4:2188983-2190473	NCBI_GNO_0400391	311617	NA	Same

7	scaffold_4:2190837-2192326	NCBI_GNO_0400392	311618	346837	4th exon too long & missing 5th exon

8	scaffold_4:2192733-2194232	NCBI_GNO_0400393	311619	NA	Same

9	scaffold_4:2194646-2196117	NCBI_GNO_0400394	311620	346838	match on all exons but NCBI model has extras at 3' end

10	scaffold_4:2634693-2636319	NCBI_GNO_0400515	311740	346840	truncated 1st exon missing last exon

11	scaffold_145:138652-140036	fgenesh1_pg.C_scaffold_145000046	115102	346841	missing 1st intron & last (5th) exon

12	scaffold_87:435725-437154	NCBI_GNO_8700117	327171	NA	Same

13	scaffold_87:437579-438974	SNAP_00023793	255735	346842	1st exon and truncated 2nd exon

14	scaffold_87:441383-442931	fgenesh1_pg.C_scaffold_87000145	111713	346843	4th intron too long

15	scaffold_40:105929-107556	NCBI_GNO_4000025	321270	346844	last (5th) exon missing

16N	scaffold_40:103864-105444	NCBI_GNO_4000024	321269	NA	Same

17	scaffold_87:211272-212862	NCBI_GNO_8700061	327115	346847	5' end missing 9 bp

18	scaffold_87:213286-214940	NCBI_GNO_8700062	327116	346848	5' end missing 9 bp

19	scaffold_87:196838-198317	NCBI_GNO_8700054	327108	346849	5th exon too long

20	scaffold_87:193344-194997	NCBI_GNO_8700053	327107	346850	truncated 1st exon & missing 3rd exon

21	scaffold_87:191113-192511	NCBI_GNO_8700052	327106	NA	Same

22	scaffold_87:187203-188649	SNAP_00023720	255662	NA	Same

23FIX	scaffold_87:185158-186582	NCBI_GNO_8700050	327104	442586	missing last 3 exons

24	scaffold_87:30125-31504	NCBI_GNO_8700006	327060	NA	Same

25	scaffold_87:31824-33484	SNAP_00023664	255606	346855	truncated 1st exon & missing 5th exon

26P	scaffold_328:49769-50798	NCBI_GNO_32800005	334296	442583	missing last 2 exons of our model

27	scaffold_4:2218191-2219707	SNAP_00002848	234790	346857	5th exon mismatch

28	scaffold_4:2216102-2217599	SNAP_00002847	234789	346858	missing 1st exon

29	scaffold_4:2213168-2214628	NCBI_GNO_0400395	311621	346859	5th intron is longer

30	scaffold_51:492193-493689	NCBI_GNO_5100060	323020	346860	extra intron within 2nd exon

31	scaffold_86:355128-356818	SNAP_00023611	255553	346861	missing 4th exon & truncated 6th exon

32	scaffold_66:753423-755119	NCBI_GNO_6600115	325025	346862	missing last 3 exons

33	scaffold_117:358469-360263	fgenesh1_pg.C_scaffold_117000028	113818	346863	truncated 1st exon

34FIX	scaffold_29:299592-300898	no hit	NA	442578	

35FIX	scaffold_123:44710-46019	NCBI_GNO_12300006	329587	442580	truncated 1st exon & missing 6th exon

36	scaffold_123:46645-48216	NCBI_GNO_12300007	329588	346866	truncated 1st exon

37	scaffold_187:187229-188772	NCBI_GNO_18700047	332335	346867	truncated 1st & 2nd exons

38	scaffold_187:180574-182181	NCBI_GNO_18700046	332334	346875	1st exon missing & longer 4th exon

39	scaffold_187:182801-184413	PASA_GEN_18700024	302748	346876	truncated 1st exon and 6th exon too long

40P	scaffold_187:184972-186472	fgenesh1_pg.C_scaffold_187000047	NA	NA	

41	scaffold_187:177577-179164	no hit	NA	346878	

42	scaffold_4:2636875-2638477	NCBI_GNO_0400516	311741	346879	truncated 1st exon & 3 exons instead of 2

43	scaffold_87:433963-435377	NCBI_GNO_8700116	327170	NA	same

44N	scaffold_6:1830849-1832318	NCBI_GNO_0600407	312608	442555	JGI – 5' 1st exon missing

45	scaffold_6:1833035-1834297	NCBI_GNO_0600408	312609	346880	truncated 5' end

46	scaffold_8:1391176-1392681	fgenesh1_pg.C_scaffold_8000220	98040	NA	same

47	scaffold_58:302684-304219	NCBI_GNO_5800046	323957	346882	1st & 2nd exons missing/3rd

48	scaffold_2:711166-709624	no hit	NA	NA	exon truncated

49	scaffold_2:705282-706818	NCBI_GNO_0200131	310197	346895	partial, last 4 exons only

50	scaffold_2:702774-704369	NCBI_GNO_0200130	310196	346897	extra intron within 1st exon

51	scaffold_2:700887-702432	NCBI_GNO_0200129	310195	NA	same

52N	scaffold_2:399077-400562	NCBI_GNO_0200074	310142	442581	truncated 1st intron

53	scaffold_13:642296-644073	NCBI_GNO_1300117	315056	NA	same

54	scaffold_138:252456-255386	SNAP_00028520	260462	346908	1st & 4th exon missing

55	scaffold_6:842460-843909	SNAP_00003790	235732	346901	4th exon missing & truncated 5th exon

56	scaffold_6:840584-842029	NCBI_GNO_0600186	312387	NA	same

57	scaffold_4:2311538-2313083	NCBI_GNO_0400416	311642	346902	same

58	scaffold_24:135381-137169	NCBI_GNO_2400021	318197	NA	same

The location and protein ID plus the newly annotated protein ID for each gene model found in the *Daphnia *genome V1.1 is given, along with annotation comments. Genes in the first column followed by the letter P indicates Pseudogene, N indicates predicted models needed revision, and FIX indicates gene models that were not initially predicted and were manually curated.

These Grs are fairly easily recognized through their somewhat conserved TM7 regions near the C-terminus, which includes a TYhhhhhQF motif in TM7. Almost all Gr genes in *D. pulex *have a phase-0 intron six codons before this motif, an intron that is present in most Gr genes in insects as well as their nematode relatives, the *gur *genes [29]. The only exceptions are the divergent Gr42–46 subfamily (see below). These *Daphnia *Grs align fully with the insect Grs, including a cluster of hydrophobic amino acids at the N-terminus that includes a few conserved amino acids. We note that several fragmentary or highly degenerate pseudogenes also exist in this genome which we have not named or included in our analyses.

There are three well-conserved and distinctive lineages within the insect Grs that one might anticipate finding in the *D. pulex *set. The first lineage is the carbon dioxide receptors, exemplified by the heterodimeric pair Gr21a and Gr63a in *Drosophila melanogaster *[53,54] and the heterotrimeric set Gr22–24 in *Anopheles gambiae *[37,55], which is present in moths and beetles as well [55,56]. Remarkably this otherwise highly conserved lineage is absent from all other available more basal insect and arthropod genomes, including *D. pulex *[56].

The second lineage is the sugar receptors, consisting of eight Grs in *D. melanogaster *(Gr5a, 61a, and 64a-f) [57-60], nine Grs in the three available mosquitoes [39], five in the silkmoth *Bombyx mori *[41], sixteen in the flour beetle *Tribolium castaneum *[43], and two in the honey bee *Apis mellifera *[44]. This highly divergent set of proteins has several amino acids that are distinctive, most prominently a glutamic acid (E) residue immediately after the conserved TY pair in TM7, although the functional significance of these residues is unknown. Three DpuGrs have such a residue, Grs55–57, and they cluster with the insect sugar receptors near the base of the tree in our phylogenetic analysis, although there is only bootstrap support for Gr55 and 56 clustering with the insect sugar receptors (Figure 1). Bayesian analysis actually suggests that these two proteins cluster within this sugar subfamily, internal to TcGr4 and BmGr7. These insect sugar receptors have a distinctive set of intron locations [29], and only the last two are shared with Gr55–57, number 2 and 3 in [29]. These last two introns are shared across the entire superfamily and hence are not diagnostic of the sugar receptors. We propose that at least DpuGr55 and 56 are functional sugar receptors, perhaps representing the origins of this sensory specificity in arthropods, from which the insect sugar receptors evolved with considerable sequence and gene structure evolution.

The third conserved lineage of insect Grs is the DmGr43a protein and relatives in other species (AgGr25, AaGr34, HvCr4, BmGr9/10, TcGr20–28 and 183, and AmGr3), however there is no obvious ortholog in *D. pulex*. Nor are there obvious orthologs for the DmGr66a protein implicated in bitter taste in *Drosophila *e.g[61], or the candidate hydrocarbon receptors DmGr68a, 32a, and the 39a protein set [22,62]. Neither of the latter two observations is surprising as these receptors are only conserved in flies, indeed the latter three only in drosophilid flies.

Instead, most of the remaining *D. pulex *Grs form three distinctive gene subfamilies without obvious relatives in the available insect genomes. The first consists of 37 proteins in the middle of Figure 1 in two well-supported clusters, specifically Grs1–29, and 47–54. A second subfamily of 12 genes, Grs30–41, share a gene structure with the above subfamily, with three phase-0 introns at the C-terminus, called 1–3 in [29], that are shared by all the insect chemoreceptor lineages (the only exception is Gr53, which lost the first of these three). All 49 of these genes also share a phase-0 intron about half way along the genes, which may be unique to these *Daphnia *Grs (it also appears to be present in Grs55–58, however the alignment is less definitive in them).

A third highly divergent subfamily consists of Grs42–46, which have a completely different gene structure, having lost all three of the ancestral phase-0 introns near the C-terminus. Grs42–44 appear to have phase-1 introns near their N-termini, Gr45 is intronless in its coding region, and Gr46 has two internal phase-2 introns.

Finally, Gr58 is a particularly highly divergent protein with a long branch hence was not included in Figure 1, nevertheless it has all the hallmarks of a Gr, including the TYhhhhhQF motif in TM7 with a phase-0 intron immediately before the final exon encoding this motif (as well as two internal phase-0 introns and one phase-2 intron). There are two fragmentary and highly degenerate pseudogene copies of Gr58 in the genome, one immediately downstream of it in scaffold_24 and another in scaffold_21. Similarly highly degenerate pseudogene copies exist for other Grs, such as Gr27 and 47.

### Expression of Grs in *Daphnia*

**Figure 2:**
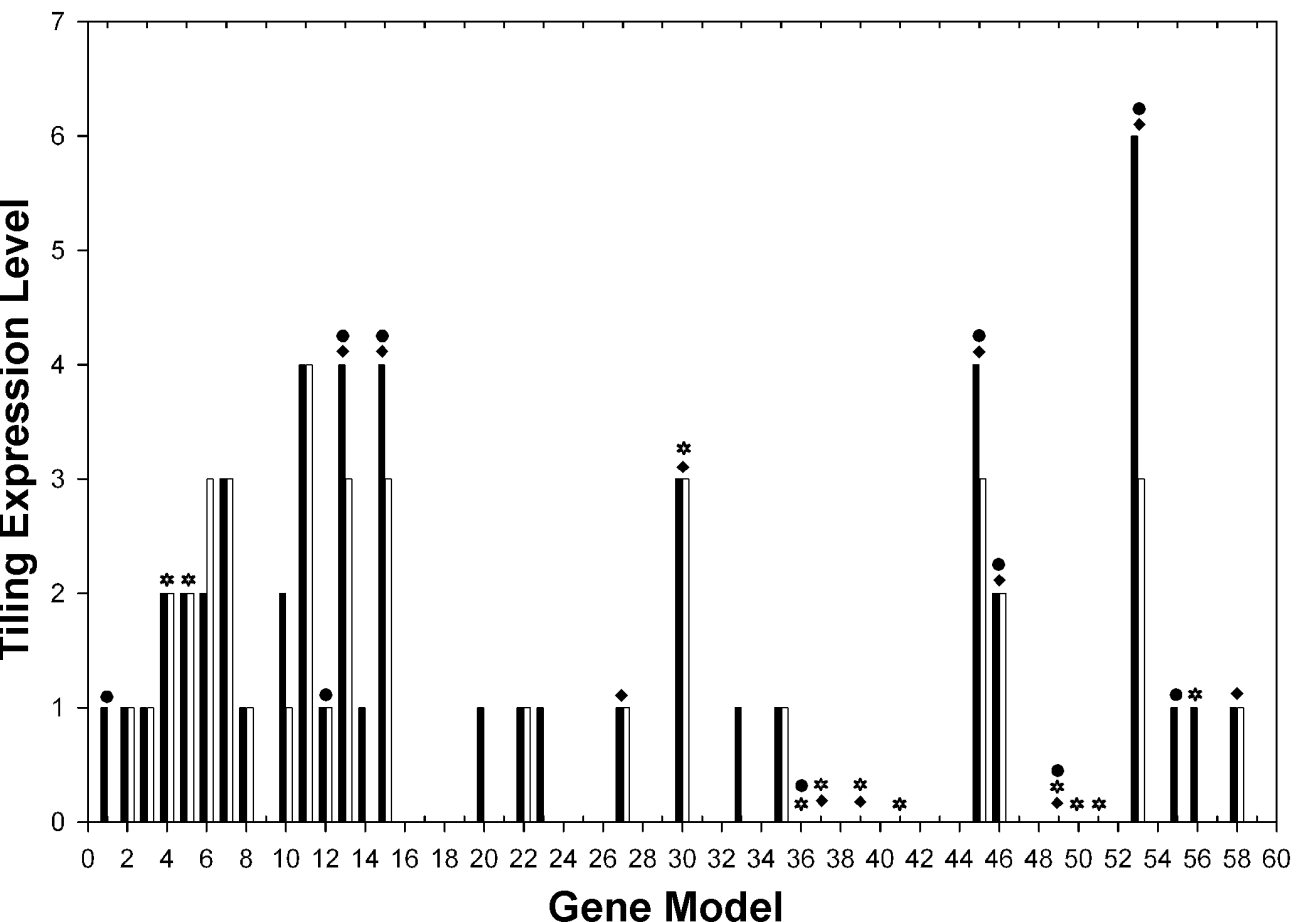
***Daphnia pulex *Gr expression**. The bars represent tiling array results which where qualitatively analyzed; expression differences were assessed based on average height of signal for each gene between the sexes. We also indicate other types of expression support from ESTs, cDNA amplification, and qRT/PCR. Black bars – female support; gray bars – male support; Black filled diamond – Genes that were successfully amplified using standard PCR techniques; black filled star – genes with EST support; and black filled circle- genes amplified through qRT/PCR.

Insect Grs are generally expressed at low levels in only a few gustatory or olfactory sensory neurons and studies in insects are largely limited to *Drosophila melanogaster *where promoter::LacZ or promoter::GFP fusion transgenes have allowed visualization of their expression patterns (e.g. [63-65]). Transformation techniques are not yet available for *Daphnia*, so we examined the only available large study of *Daphnia *gene expression, an unpublished Nimblegen genome tiling array experiment comparing males and females using whole bodies, performed in conjunction with the genome project (J. Colbourne personal communication). This reveals generally low but convincing levels of expression for 27 of these genes (Figure 2). Gr11, 13, 15, 45, and 53 show particularly high levels of expression, of which all but Gr11, have female-biased expression. Only one slightly male-biased receptor was identified (Gr6). PCR amplification of a subset of Grs from female and male cDNA supported expression for 11 genes and some showing negligible expression on the tiling array were also verified using qRT/PCR amplification from whole bodies. This investigation revealed that 7 genes having negligible expression on the tiling array, are indeed expressed (Figure 2). There is no obvious pattern of expression level with clustering of genes in the phylogenetic tree (data not shown).

## Discussion

We describe the 58 Grs we found encoded by the draft Daphnia genome sequence. We believe these constitute the entirety of the "insect" chemoreceptor superfamily in *D. pulex*. This superfamily of odorant and gustatory receptors was identified originally in *D. melanogaster *and has been identified in all other insects with sequenced genomes, and it was anticipated that it would also be present in other arthropods. The absence of the Or family, a single particularly highly divergent and expanded lineage within the superfamily, is consistent with the prediction of Robertson et al. (2003) that the insect Or family evolved with terrestriality in insects or their immediate hexapod ancestors, although sequences of additional crustaceans, other arthropods, and basal hexapods, will be required to test this hypothesis further. We have undertaken several steps to identify all members of the Gr family, including highly sensitive TBLASTN searches using only the somewhat conserved TM7 region of these proteins, and HMMER searches of all available predicted proteins using all available Grs in the model set. Grs can sometimes be extraordinarily divergent, however, so it remains possible that some have been missed. For example, Kent et al. (2008) report five new Gr genes in the *Anopheles gambiae *genome that were missed by Hill et al. (2002) because they are so highly divergent and automated gene models for them were not sufficiently well built to find them using PSI-BLASTP searches.

The only *Daphnia *Grs with a clear relationship to particular insect Gr lineages are Gr55 and 56, and perhaps Gr57, which cluster with the sugar receptor subfamily. This indicates that *Daphnia *likely can sense some sugars, presumably dissolved in water and perhaps indicating food sources [66]. Despite extensive searches we find no orthologs of the other well-known and highly conserved Gr lineage in insects, the carbon dioxide heterotrimeric receptors, represented by DmGr21a and 63a [53-56]. This is perhaps not surprising given that *Daphnia *are not known to be able to sense carbon dioxide, although it appears that *Daphnia *epphipia (or resting eggs), do respond and at times require a carbon dioxide signal to hatch (see [67]). The only other relatively well conserved Gr lineage in insects is that of DmGr43a, AgGr25, HvCr4, and AmGr3, however the conservation here is insufficient to expect to find this lineage in *Daphnia *(Figure 1). The remaining insect Grs for which ligands are known, DmGr66 for caffeine [61] and DmGr68a and 32a for cuticular hydrocarbons [22,62], are dipteran-specific lineages, hence were not expected to have *Daphnia *orthologs.

Instead we believe there are only three other major Gr subfamilies in *Daphnia*, all expansions within crustaceans, consisting of 37, 12, and 5 genes. The highly divergent Gr58 might represent another subfamily that may be more evident in other crustaceans.

An interesting feature of some of these *Daphnia *Grs, e.g. 31–34, 36, 37, 39, and 41, is that they end immediately after the conserved TYhhhhhQF motif which forms the core of TM7. These are the shortest versions of Grs known, and indicate that the C-terminus of these proteins is unlikely to be involved in any important interactions with other proteins. This situation is compatible with recent findings that the insect chemoreceptors likely have the opposite membrane topology to the TM7 GPCRs [50,52,68], because the C-terminus would be external to the cell where no significant interactions with proteins in any signaling transduction machinery would be expected. They therefore support the hypothesis that these chemoreceptors are not coupled to G-proteins and instead function as ligand-gated ion channels [69,70].

## Conclusion

This repertoire of 58 Grs presumably underlies the many abilities of *Daphnia *to sense their external chemical environment, which they do using both a classic "taste" mode involving physical contact with objects, as well as what might be considered a "smell" mode in which they sense dissolved chemicals in the water. As elaborated in the Introduction, these include food, potential mating partners, and potential threats like fish. Therefore, we suspect that these genes will be expressed in identified chemosensors, such as the first antennule and feeding appendages [71]. Our preliminary assessment of expression levels of these chemoreceptors comparing males and females reveals apparent female-biased expression for a few of them, but no clearly male-specific receptors that might perceive sex differences. The next obvious step in studies of these *Daphnia *Grs will be to determine their expression patterns more precisely. Initially this will be achieved by RT/PCR studies of surgically separated structures, like the antennules, although this is technically challenging but achievable for such tiny animals. While in situ hybridization might allow more refined studies of their expression patterns, in *D. melanogaster *at least, Grs typically are expressed at too low levels for reliable in situ hybridization. Ultimately studies using promoter::GFP fusion transgenes might be required to establish confident expression patterns once transgenic techniques are developed for *Daphnia*. It will be of particular interest to determine whether any of these six gene lineages, for example perhaps the most highly expanded 37 and 12 gene subfamilies, is exclusively expressed in the antennules or swimming antennae, in which case these might constitute the effective "olfactory" receptors of *Daphnia*.

## Methods

Known insect chemoreceptors whose sequences have been entered in to GENBANK (National Center for Biotechnology Information) were used to search for similar genes in the *Daphnia *genome sequence. Protein sequences were used to perform TBLASTN [72] searches of assembled scaffolds available through two websites: Joint Genome Institute (JGI) *Daphnia pulex *V 1.0 and V 1.1 [73] and *Daphnia *Genome BLAST [74]. Genomic scaffold sequences were used to constructgenes manually in the PAUP*v4 [75] and MEGAv4 [76] text editors, using comparisons with known exons and online programs to predict exon/intron splice sites [77,78]. Divergent *Daphnia *proteins were used in iterative rounds of TBLASTN searches to find additional genes. In three cases genes were truncated by the ends of contigs, but in each case the complete gene sequence could be assembled with the aid of raw reads, and these are indicated by the suffix FIX after their names. Two genes in the named set are clear pseudogenes, with internal frameshifting deletions, and are indicated by the suffix PSE. All proteins were aligned using CLUSTALX [79], and gene models refined to fix apparent alignment difficulties. Intron locations and phases were located in the alignment in the text editor of PAUP to assist gene model refinement and subfamily analysis. All proteins are available as a FASTA file [see additional file 1].

Our manually curated gene models were compared with the set of 30,907 gene models generated by the JGI known as v1.1. They were also validated through nr, SwissPro and Pfam hits. In summary, 13 gene models were identical, 13 needed minor revisions, and 29 needed modification, and 3 (Grs 34, 41, 48) were completely unannotated. 44 genes genes where supported by nr, SwissPro and Pfam hits, with the drosophilid Gr64 sugar receptor family supporting DpuGr 55 and 56 as potential sugar receptors. We also compared our gene models with preliminary tiling array expression (NimbleGen, Madison, WI) results to see if expressed exons agreed with our predicted models, and 27 gene models gained additional support thereby.

For phylogenetic analysis, representative insect Grs, primarily from *Drosophila melanogaster*, *Anopheles gambiae*, with a few from *Bombyx mori*, *Heliothis virescens*, *Tribolium castaneum*, and *Apis mellifera*, were included in the alignment for comparison. The length-divergent N- and C-terminal regions, as well as internal regions with major alignment gaps, were removed, leaving 328 aligned amino acid positions. For the main phylogenetic analysis, corrected distance was performed in PAUP*v4 using the heuristic search with tree-bisection-and-reconnection branch swapping. Distances were corrected for multiple amino acid replacements in the past using the maximum likelihood model, the BLOSUM62 amino acid exchange matrix, and default settings in TREE-PUZZLE v5.0 [80]. Additional Bayesian analysis was performed using MrBayes v3.1 [81] with the JTT substitution model, four chains, 1 million generations, and two runs. Trees were sampled every 100 generations, discarding a burnin of 250,000 generations.

Using the polymerase chain reaction (PCR) technique we designed primers for assessing expression of a subset of our gene models. This subset included genes having EST and tiling support as well as those lacking any type of support. Primers were designed and tested on both genomic DNA and cDNA of *Daphnia pulex *male and female clones. Quantitative real-time PCR (qRT/PCR) was run on a few models to assess differences between the sexes and to investigate whether lack of support was due to low levels of expression which standard PCR cannot amplify to detectable levels on a gel.

## Abbreviations

Grs: gustatory receptors; Ors: olfactory receptors; ESTs: expressed sequence tags; PCR: polymerase chain reaction; qRT/PCR: quantitative real-time PCR; JGI: Joint Genome Institute; DOE: Department of Energy; TM7: seven transmembrane domain protein.

## Authors' contributions

HMR and DCPA annotated all genes and wrote the manuscript. DCPA performed all expression analyses. HMR performed the phylogenetic analyses. ML supervised and helped edit the manuscript.

## Supplementary Material

Additional file 1 ***Daphnia pulex *Gustatory receptor gene models**. FASTA format of all 58 DpuGr gene models. Click here for file
