# Supplementary material for: The chemoreceptor genes of the waterflea Daphnia pulex: many Grs but no Ors
Source: BMC Evol Biol. 2009 Apr 21;9:79. doi: 10.1186/1471-2148-9-79 (PMC2680840; doi:10.1186/1471-2148-9-79)
Supplement: Additional file 1 — Daphnia pulex Gustatory receptor gene models. FASTA format of all 58 DpuGr gene models. [file 1471-2148-9-79-S1.doc]

>DpuGr1

MFYVRPTGAVKTIPLKDSKMEDKNNVKILKMRKNSFNWSLEPITKCMHVMGVSIPPSEEIKSCRLICHLYRTFCFLLVSFVQIILVIQVFGDAKNIANSYTNGISTTTFSWNFIIDNLNLALYTVGSHTSFLLLTRPKTWLDLVLSFKLLEENGHSSEIYPICRQTAVKAVICIITMNFGLDVLLGLDVLSNDAPWARKILDVLGLVNKIYPAVQLALFSILMKMISLQLKAIRKHFQDQFTQSHPSSLMLFESQRSHLIALRGHHRLICNAIRKLNRHFGVFLTLEIIFIFVSVINCSLFVLMGATSGDGLLGGLNAAICLESLVHLLILSSFSDDIPNQTDKVYEALLQLLHHQPSLQNEVSLFTEQLLHMKPQINAMGFFIVGKHLFPTLIGTTLTYFLILVQFHSAEKS

>DpuGr2

MQEICQVKVQPTQSSNLESNGVKLGQRSSFHWSLEPMTKWMCLMGVPISMNRSSCQLSCCRFNRIFCFLVVLFIHSSQIIHMLLNAKSIANSYITGTSSTALSWNFIIESAIIAVYAIGGHISLLLVTRSETWMDLVHSFKFLEKNLTSSDIYPACRKLAIKTIIYVISSVLCIDLLLGIDMIWNESTLMRRILDLSSFFSKIYPMTALGLFCILAGLISLHLEVIRKRIDDQLNQCKHGENFSLVIKEQRSQLKLLRRHHRLICNLVGKLNCCFGFFLLVEVAFIFVTSVNCFMYLFSSATRSDKIMGLLYSVICLDSLVHLFLISSFSDDIVNQTKKIQEALVELLHDQPSLQNEVILFTDQLLLLKTHVNAVGFFNVSKHLFPSLIGTTLTYFFILLQFHSAEN

>DpuGr3

MSHYGLVKVKPIGLNESEQDVKISTSEHRKILWSLEPLTRWMWLLGIPIPPSLEDSDCRLPSHLYRIFFFSMVLLIQSSQLIHIFYYAEDVSMAYMSGLATSALNCVFVIENFCLAVYIVGSHICFLLLTRSKTWSDLIHSFALLEESILCSDKLFISCRKLAVKLIIYLIFSFVWVAALLVMENIYNSNSVTRKMMDVSGFLTKIYPAVQLVLFCTLTGVVSHQLKAIRKRISSAPTSVEAQRSQLIALRNHHRLICKTVRKLNRYFGVFLTIEVGYVFVISITSSMYILLGGMSIDGLLEALNVSVWIDVFIHLFLLTFFSDDIVSQTDKVYEALLQLLHQQPSLQNEVSLFTEQLSHMRPQISAFGFFAVGKHLLPSLIGTTLTYFLILLQFHSAEKPS

>DpuGr4

MLVPTRTVSEEAVEKGWLWSVQPLVTWAKWLGIDLSDLSSGGRKKVSRWFVLYSVFVLLISVSLQLPCLSYIINNHKEISVTFTIENSYNSDTFSWNTAMDYVNFAVHSLATHVIFLSVFRARWNLLGETFQNLESFLKFSFFDRIRKASILGLVWIILLHTTLLATNLYHHSEYGSSFLILFCSLVSVFSQVYPITAIVLFSAVCYASSNAHQSIRMELNLLKNTTTDGNYYNRLSTLKRRHALVCETVDHINHCFGFFMGISLSFHFVSMITASFYLFGTDKEPGSVLEIGFACSQICNLSLICYPADLIRLKADSVFRKLIRMQLEMEEPLKSLTNAFAEQTSLFFPQINAAGFYPISRKIIPQIVGTTLSYFFILYQFQSAERSDTSVNAGI

>DpuGr5

MPIRAATVGANQETSETIVSVNNGTAGKYWNWSMHPLIIFSMLIGVDLTVPSKNSCRQKWKKIFCVISLMIHAAFTLNLVQIFQGQLSEMTSFYFGEDGNQSTTALWNVIIDVINFEVGCFGAHWIMYFFIKKRWASLCDAFQLLVPLFDAEFYKEFHAKLRRCSYYGLACIVLGVGSLSIIGVVPNINTETTFNIKFMALSQAFFQIYHVLSITLFAIASYASSISFQSIALKLNGWKNPQALDLRPNADLILLKRYYVLTCQTVDSIHHCFGWSLLFIVPHLFVAIINASFYWFAKTNENNSASEIAFFFFLLINLFVVCTTSDRVRIQSERVLKELIRLRIEPRHNHCRKQVHELLMQVVLNSPRISALGFFNVSKKLIPTMIGTTLTYYFILRQFHSSEKKSFR

>DpuGr6

MAIRVGLSLDEPQVEQTKCWAWSIFPLIIWTRILGVNLSDISNSSAKRHHWLIIAYGSFCLLCHLAGQIDILYYLQGKLKMGSLERSGGLNFETSTATWNSIIDFINYAVHGIGTHVLMLTVIRKRWINLMETFRRSEYMFSDERYIRIRKVATYGVAYVILLILGLISVSLGHHLAVGTTSGHRIFISFMSTVSIIYPLTALVVFAIHCYVSSLLFESVQSEIHRNEIQLMNGRDQDVRLLMLTFKQRHILACKTVDSINHCFGWILLLSIPFFYLAVINSSFYLFGLDNIISMPDIAFTTFTVIHLIFVCFTADRVQIKAEDAIKELFRLQSHETLNGSQMQLMNSLISEMALTVPKISANGCFNVSKKLLPQVIGTSLTYFFILFQFEASEKNVTLTEN

>DpuGr7

MSTRTSSFSEETSEQQIIGWAWSIRPLIIWARILGVDLSDVSPSSDSHQWLTVAYGIFCFLLHISGEINSIYYLYTTQTVHVSLEQSYGLLFVTTTATWNWTIDFINYVAHGIGVHVILVTVVQAQWKDLMKIFQRLQEEFPDANYIQIRRLSLFGVAYVILMISGLVAASVTDYHLAAGSSSIHSIFITITSTLSIIYPLTTIVLFAVHCYASSVAFELIRTKIGRGRHVRILLLTLKNRFILASDTVDGINRTFGWMLLLSTTFFFVAIINASYVVFSLDKQITAGDLVFLLFALVHLTLVCFAADHITINAEEMIGDLLRCKCDKMNKEYQIEMEYLISDMLHEIPQISANGCFNVNKRLIPQVIGTTATYFFILCQFEESEKKVCPVS

>DpuGr8

MAIRVSWFSEEIPIIQQPATNWWAWSIRPLIIWLRIIGVDLPDTSTPSTQRNRWHISVYRAFCFLLHASCQINILRYLFNTGLQVFLYQTDDLGFGTTTANWNWIIDFTNYTIHGVGIHLVLLTVIRFQWIDTMEIFQRLNAIFTEENYIGIRKISYWGVIYVIVLISTLLAASADYALPSGTSLSHSVFIKMTAVLSITYPLTAIVIFAIHCYASSLAFEGIRKEIKRYEIELINGRNHDVSLLLSVFKQKHIIACDTVDSINHTFGWMLLFSMTFFFVVVINASYAVFGLPDHTTVADISFLAFALVHLPLICFAADHISNKAENMIKELMRLKFADEPLKGYELEVEFLIVDMVETIPQISAKDYFRVNKRLFPQVIGTTLTYFFILHQFKASEKKLDPEDDESY

>DpuGr9

MATQVSSPKDQTKDRQTLPTAECWEWSIRPLILWIRILGVDLPDEDYRHHRWWMYVYGGVCFSSHAISQCVILHFVLYEWKQVNNQDSGEFIYGTVTATWNWIVDFMNYAIYGVGIHFMLLTLIRLRWNRLKKTFQQFQIIFTEENYVRIRQISSFSVIYAILLILGLAVVNLIQQARQGASPRRIFITFMSTLSVIYPATALVAYTIHCYASSLAFELIQKQIELFQIELANDRDLSLFLQTVKRKYIAVCESVDAINHCFGWMLLLSTSFVFVANINSSFYLFSIEININDFAFLSFSLIHATQLFFSADRIRCKAKDFLQELLRLTCDEKFKEFQLKMKFVFLDMVRSIPQISASGYFNISKRLFPQLIGTTLTYFFILCQFQASEKNCNLTK

>DpuGr10

MCALGHLSTDIINQSSQQVAALLPTVERNMLRKRVARQVSCSSDEETMMSFKWCCRPVLIVTNIFGIPLKMKEDEPSYSAWMVYIFGWILYCTNVAIGLIIIILCQDAGNVSSLSPDSEEFNNSTTASRWNEGISTYNTISSMIATHTVLLAVTAVRWEDLVRVLDRMERTNQFNLKEFSVFRAIFHVGFVFAVIGCLATGLLSISTYFTIDLQLWQMLIFAVQRWLILYILLAVLLFTCFGWMAATMLQMVGKEMELIQENGQLHENCDGPVDVLISKCSRHYFVTIDFIHQFNGCFGCLLLVLIAPAFIRIINASFQLMINLKDGHWTMDMIVQFMILLVHCVLFTLVANIPHKIRQETFNLTKKLRKLYFDDFSLQNQVNVLIMEISNSVPKITAAGFFDVDLQLIPTLVGTTLTYLIILFQFQTSEKN

>DpuGr11

MNEENLEWGWKPILIAMRFIGFDLRDPLVSPKNSWFNFYSFLCLVYTAFWHVFDCVDTTKFFMRVLDSDPPSNISLVVITNVMVDTYNNAFHSVGIQLTVYFMARSNWKEMWQVVQRVGRRFDFKFYENLRIVSSSAVVAVVVIELYLLASGIVYTALSPYLTFVLHGKLFALILAEIYRICGSSLFIVCALSASDVFSQLKNEIDKASRYLNPPDASSILKWKHTHALACVYVDSINRCFGPILLIDICCIFLRMINSVFQVSVGLNVHKSRSSIYHTLYSLELFFQFWIVCIVASRLQIKATNIIQTIRKLPTATLDTQRLAGTLLIQVHHSAPSISAYGLVKINHKLVLKLIGTTFSYFIIINQFYLLEDPLQQ

>DpuGr12

MLRRLVDKVLDSFKDETINFEKCFRPVLIAMNMCGIPLGMNNTDEPTTLTVWMVYIFGWILYCFNVASGLMSISIEKREKSPFFTNNRTTAWQWNYGITTYNSVFSSIAAHTVLLAITTVRWKHLARIFHRMERINQFGLKDFQAFRAIFRSGLFFAIISWLAIGYQSISAFFMANVDVWKMVIYSFQRGFIIVIILAGILFFCFGRMASTMLQILGQEMAKFISSDDDQQSFENIDVVIAKWNRHYFMAIDFVHQFNRCFGCLLLVLIAPIFIRVISTSFFLMIELKDGQWTAAVTLNLIVLLVHFVAFIFAANIPHRIRQEAIDLTKKLRRFQLKDFTHQNQVNVLMMEISNSLPKITAAGFFDIDLQLIPTLIGTTLTYLIILFQFQTSENT

>DpuGr13

MSFQWSLQPMIKWMHFIGVYLESPGKDNSSRFRLFVTAYGFILFFVNVYSNGMMAAKFLQGFENISHQTTSSVLWNAVVSQTNYILMTIGCQLSLISGAVSTWPGLIHILREMEKERYFVSKDFHHFRRIYLSGLGFLIAVILAILGIAVTIIMSTQNQTKVDTLFQICFTFLLIFVFSGGALFSCFGWMVASMLTILAERVVEQSNSNCSGCDWEKTFVRWRHLYLKISRLVDKMNECYGFLLLFLITSSFVVTINSSFIIMRDVNDEGIDYNCFIHSFFLIIQFSHFAVLAYVPHRIRESAIYLSKQLRELKIGNEARQNKVNFFILDVLNSLPQITALGLFDVNLRLAPTIVGTTLTYLVILCQLHYSAK

>DpuGr14

MVKSSRIKVVARQTLAVVSPSNIVTSDCLTNDSTVLNFDWSLKPITTWLQIFGIQPPLISPVNRDSCCNRFVLLIPIAAVFYFLNVGCNGFALGQMKWNFIKKNYSSSTRNWNLIFNEMNFSFLTLATHSALLFFSYFMAWNGLTEVLRRLEQCHQFDSGHYKKFRKICTVGSCFMLIEIAIELFETLYGHLLFDAKILPTFRVILSGLRMVSLVLPFLGTILFCCLGWMASIMLRIMAEEIESSIINRPDYNSRLNNWVLMSLDGWRRRYFLIVEFVQQINCHFGFILLVVTASEFVRITNTSFQLLIEFLNNSWTLSSMSTILIAFDFIKETACFCILLYVPTRIHGEASDLVKQLRMMDFDDYGPRTQIKFLVEEMTHSLPSIAPMGLVQINVQLIPTLIGTTLTYLIILCQFHSSETV

>DpuGr15

MRTMASVRTEEPLPPNIQDCSVVRNNDDPVDGGYKNPSPFQPLVVFLRFLGIELDPALLYKSSKCFRYLLLGLCFLLLNAFFTIFTAVSEHNFFVQIYNMDTPTNSSSVENNSTVPSVFLWNLIVDYVNYGVLSVGVHASLLFFSRQKEWKFLWDNVHQILQHHNEFKGIWKSIRRVTIAGLLIACLETLGFISCSLVLIDFNEGVTLEFYFRPIANFSYVYATCGLLLFAIIGWTVTLGFRCLHQELLSNCLLITCNNNGCPKTVSLAEKEELIEMLANWKQLHVLLIDTVDGINDCLGPVLLIWVAHIFVGFIATPFYILDGIHFNTGSLSSTWVFLIMNFCLFAQHLFHLLIITGISSRIWHEAAANGKLLQKVNLNDFYHLQDQVNMLTTEVFLSLPRITAMEYGDLDFSLIPTVIGTSLTYLIILCQFQSSEE

>DpuGr16

MASVRTDESLPPHLPAWTVELNNADDPVDEARENPLPFQPLIVCLRFLGVELDPAILKKSSRCYRYLSLLLGIFLLLLNGLCNIYILILNQHQMAGNQNESDESEVLSVILSSANDNYQPITESSTMSWNFIMDYVNYISLAIGVHACLFILSRQPKWEFLWDNVQQILQQFNDWKKTIRRVTIAGLFIISSEISIYVGLSFGFVELTNDKQLFRDLFICCFVNLFLFYALSGLLLFTLIGWTVAMGFRSFGQELISKSLLTNTRSNDDASLDGFRLMIIKWKKLHILLIDTVDGINDCFGPVLLIWVSYIFVGFIASLFYIADEVYFNSTLSYSSWVMLIMSSVQVAQHILHFLIITGVPNYIRREAINLGKLLKQVNLCDSHLQEQVNVLTTEVSLSLPRITVMEFGDLNLSLIPKLIGTSLTYLIILCQFRSLEKI

>DpuGr17

MGAMASVRTDESLPRRFPAWAVDEGNKGDPVDTARQIPLVPFQPLIVCLRILGVELDPAILNESSKCYRYLSWSLGLFSLLSNGLCNIFAGVLGQRKNIYEHNKAVLSTYNPSLIDGNNSTISSVMSWNLIIDYVNYGVLAFGVHASLFVITRQQKWKLLWDNVQQQILQHHNEFKEISKSIRRLTIAGLLIVILESLWLASSPLYFMRLDETHQVLVVIFSKSFSYLFHVYAFCGLLVIALIGWTVAQGFRSLHLELKISSLFTTASDIRPALEKEEMIQKMLTDWKGLHVLLCDAVDGINDCLGPVLLIWVAHIFVGFIATPFYIFGGFRSSQTTNTTIIALNLSLMVMLSFHLFVITGIPSRIWHEAIENGKLLQQVNLKDSNHLQEQVNMLTTEVFLSLPRVTAMEYADLDFSLIPTVIGTCLTYLIILCQFQSSEE

>DpuGr18

MRVMVSVRTDESLPPCFPAWTVELNNGDPAAVDDEAHENNPLVPFQPLVVCLRFLGVELDPAILNGSSKCHRYLSWSLGLFLLLSNCLCNIYNAILTRNQIAGDENATVFLLSSIDGDQHLPITKSTTMSWNLIMDYVNYGSLVIGVHTCLFILSRQPKWKSLLDNVQQILQEFPELNKTIRRLTIAGLFIIFSEISFYVGLSFSFMELNSGDGQLLHDLLFGCFGNLFLFYPLAGLLLFASIGWTVAMAFRSFGQELISKSNTRSNDDEASLNDGSHLMIAKWKQLHILLCDTVDGINDCLGPILLIWVSYIFVGFIAALFYLADEVHFNSALLLYPSWAMLIISTVQVAQHLLHFLIITGVPNYIRREAINMGKLVKKVNLCDSHLQEQVNVLTTQVSLSLPRITAMEFGDLNLSLIPKLIGTSLTYLIILCQFRLLEKN

>DpuGr19

MLLKIEKSLWPLTVWMKIFGFPMEIPTRKRKSGSKNYSICLLLTGSLMLLSTVALHCTSFVHGVLRVNKLGPSSNDGNNLTTANLLNAGIEHLNNTWFNIGVHSTFFLVSLTSNWKSLWHSLLLIEKNLKFKSSFYRKCRESVSIGFGCLLLDFVTHLFISVRSSYWDLGVMRSLAVVLANFSRTTISSVFLLFCVTARVITLVFKALNKQIVNLTEAEKILPIYLSARILNLRLERWRRNHTLACELVDMANKCFGLVMLVTIINVFVSFVTTSFEIVSSMGNHESLPFSLLLIFVNKSILLAIVFFESYRLQSEAGRTAASLRKLHPLTADLFTQIKVNTVVMEVTHASPKIAAMEFFDVNIRLLPTLIGSTLTYVAILHQVAAHSK

>DpuGr20

MSGTANVCEVRTGRTDIFCRMLCVELILSIGQQHAHQCVFIYLLVQVVPFLSGRSRLPEMLKIETSLWPLIVWMRIVGFHMGPSRKKSGSPNRHRYSVCLLMTGSLMLLSTVALHCTSFVRSFLKFRANGMAQHNGTTANLTTANRLNVGIEHLNYTCVLIGVHTTFFFVSLTSNWTGLWDSLQLIEANLKFNCTFYRKCQKCVVIGFVILFVDFASQLFISIHSFYWDMGLMSPLAIVLANISRTTISSVFLLFFVLARVITLVFQGLNEQIVHLDEVEQVPQCYSVSRILNVRLEKWRRNHTLACELVEMVNKCFGFVMLITLVNVFVSFVTTSFEIVRSMEDDETLPFLLVFIFVKKSILLTIMFYEPYRLQAETGRTASSLRRLHPFTADLFTQIKVNTVVMEVSHACPKITAIEFFDVNLKLLPTLIGSTLTYVAILHQVASHSK

>DpuGr21

MLNLEKSLWPLMAGLRLLGFHMGPSSSSSSRYSVCLLSAGSLMVLSTVALHCTSFVFGVLRLKANEIGPNGANLTTTKLLNIGIEHLNYTWVHIGVHVTFFFVSLTSGWTQLWDTLHLVEENLKFNATFYAKCRRSVLIGFGFLFMDCVTHIFPSIRSFYWDMGVMSPLAIVLANFSRMTIISVFLLFCVLARVLTLIFQGLNEQINDLDEPEKFSSVYSSRILNLRLEKWRRNHTLACQLIEMINKCFGLVMVITVVNVFVSFITTTFEIVNCIQESDTIPVMFVFIFAKKSCLLTIIIYEPYRLQAEVSRTAVALRNIHPLTADLLTQIKLNTVVVEVTHASPKITAMEFFDVNLRLLPTLIGSTLTYVAILHQVSSHSQ

>DpuGr22

MLLSIETSLWPLMVGLRIFGFHMGPSQKSGSSRYSVGLLLTGSLTVLSTVALHCTSFVYGVLRLKANGLGPNGTNLTTANLLNIGIEHLNYTWVLIAIHTTFFFVSLTSSWTELWDSLLVLEKNLKFNSTFYAKCRKSFLIGFAFLLMDCLSHLLVSMRSIYWDMGVMSPLAIVLANISRTTIISVFLLFCVMARVITLVFQGLNEQIARIDEAEQFSPFYLTGRIWNGRLEKWRRNHALACELVEMVNKCFGVVMLVTVVNVFVSFITTTFEIVRSMQEDATIPVLFVFIFVKKSILLSIIIYEPYRLQAEAGRTAVSLRKLQPSTADLLSQIKLNTVVIEVTHASPKITAMEFFDVNRRLLPTLIGSTLTYVAILCQASSTHSTFK

>DpuGr23FIX

MSATISNNENEFYLTCFKPLAVWMRLLGIELDPFDLPKRRIFCSRSFSVLMLSMTLLTNLAFTTITFNQQILSILPSSNFKGSASDFWTLVIDFGSFAVLAIGAHATLLAIPQNSEWRSLWTNLQQLATENGPKLFYSKSRKTVLMSLLFIIADGCNNTIQGVFTALKDDGRPTLTKLADFFSFLTQIYGVSGLVLFATIGWITSEGFVQFRYQVIALDPAIHPNPSVTTSDLKKLRRDFLTLSEIVLHIGNCFGPLLFVWIVYIFISVIANSFYLVNSVRIQVEHLSEVPSWISLLTRNLIFLFVLTSIPVTLQRESLNTWKSLRRVSFDDPSLQCQLDTLTMEVLQSPTEMTAMDLFQVNLTLFPTLVGTTLTYLIILSQFQSK

>DpuGr24

MRSSDLPNEGGQLDHRFQPIFIWMKILGISLTGERRFMGNVYDLLMLAITLASNIASITLMLVEPERPKQEDLSIITSYTFTQILYVDNLNFILLVVGAHLMLLCQTRRHWKKLWATLLEVQVYLNRKILNRSYVIALLLIPEAIMIVLVVPLDVIGLPLYQQLVEVVSCATKIYAVSGMILFCMCGWIASDLFQVLLKQLNDNPSNSSMCLKTVKKRYHQVCQLIDDINQCFGFIVLVLITYMMASVVMVCFVAVVEFQHYDSTAVPWLYVRDWILLIQHVVNLMAITYIPHTINVQNQQIHLALGRIEIDSRVDSSRHEQLEFLKWDVSQSSVKLTAAGLFDITPRLFPKLIGITMTYVIILFQFQSSEKTT

>DpuGr25

MYCITVAYSPYILALYTIRGGEQLRLFNCPVDMSPTRKINVSIRKDVDSFNFTWSFHPIFVWMRTVLGIELNDNRLVLLATADSAVEPLRRRCCRWIRRLFWGFLVLVISLSVNILDAIFTHDDTIDSNYLLHEEKVNVTVSDLMIIRIAAVNEVFYNGTIHLIFYTLACCLDQWKHLWTILQKIQFQLPKLDREFYFTCRKIAVVGTICIFVNCGLNVRLAINNLRTTLPIDVVILVTSLSKLSETLSLSILVLFFVLVWSTIHLFRFVNSKTAGLLTPTDSSSNVPISFTVSQQLENWRRLHALTCQLVDGINSCFGFILLVAMTHGFISFISDSFEITMAFSNGSNFQTRFLIRFIQHFALLFVVCYSSYRLQAEASNTSKCLRQFNFSSVLPNLHIQLNGVMMEVGQTQSKISAADFFDINLSLIPTLIGTTVTYLIILYQQANAEAK

>DpuGr26PSE

MFSVEESLWPLIVWMKIFGFQMGPTKKPASISSNRQQHHQSICLLLIGSVMVVSTVALHCTSFVRGVMRLKANGLGPDGANLTTANLINVGIEHLNYTCVLIGVHAGFFIVSLTSNWSAVWDALILIQENLNFKSSFYHKCRKVVLVGFSLLFTDCIIHIFVSIQSSYWDMGVMKPLAIVLANISRTTAMSVYFLFCVFLPSASRGIXAGRTAASIRSLQPLTSDLFTQIKLNTVVMEVTHAGPKITAMEFFDINLRLLPTLLGSALTYVAILCQASST

>DpuGr27

MTRLFRNFIRVRTRSQSELVLFPAMEGNKVSPSAVQESNNGNVSWCFQPFFIWMRLLGIELYHKCFRFSKFINVYGILLLLMCEWASVDFIAIKFWKISFPFTQQQSTNNALNVSMSTTLSWNARIDTLNHFCFIAIVFPTFFHMAHHKWLRLWNVMETFDFAYCKLNHDKLRRILLIGFFPILAEIGIIIYNLLVKIPDFANETSYRKFVTATAYFAKIVPISALVLYCSVVWLSSLFYEEIRLDIERTYKVNQENIRRWKCSLVLAGEVVDRINDTFGLILLISITHFFVEFIARSFYLVNSISTTNRPDCITLVSMSSEVLFLWFIAYSPAKIHHRAIQVGKSLRKIDSVDNNLQNQVSLLALGIVQNLPKISALGHFDVSLHLIPQLIGTTLAYLFILYQFHASERND

>DpuGr28

MVVTKVVPVVPVVPPRNNISWCFKPLFLWMRVLGIELDPVSNPYRQITFIYGLIMLLLCQWASINLSANSNIFIVSSPITARSNNDSNISTSTTLGWNLKIDIINNFFLMAIICPAFFYIAHSQRWLRLLDVMKKFDFAYCRLNRGERVRHFVFVGFVPILSEIGIMIYNFKTSIQRVPNASAYQIYLISFAYFAKILPISALVLFGSVVWQSAAFYGEIQQDVERNLSSKLKKENIGKWKRSLTLVDELVDRINECFGLILLVSIAHFFVEFIARSFYIIINCFLRNQLDLINMITMSSRIFLLWYFVCCPAKVQDNSVQVAISLRKVDYVDINLKNEISFLTTDIIQNLPGVSALGYFEVNAQLIPKLIGTTLTYLVILCQFQSSENKD

>DpuGr29

MHLVVRSPLKNNFIATDTVAGIPWCFRPLFIWMRIMGIDLYHPTSRKSIRRYGLVMLLLGTWAHIDNNLDTFRKLLSNKLSASATLDWNIGIDYVNNFGFIVLVSHQIFYVAGNQWLLLWGHVVKFDLDYCRFHHKLLRKLLLIGFLSPLLEICNTLFIFSSILPDFAKDSTYRKCITVLAFFSKILPISALVLYCSVAWLSSLFYEEIRKDIYYTKVINEKNIRKWKCSLVLAGNVVDGINDTFGLILLISVTHFYVDLISHLFYTWNNISSLTRLGATIYLVNVLRKLVFFWFIVWTPTEIYRKAFKVATLLRKINGVQFHLQKQVHFLALDTFKNLPQISSLGYFDVNLRLIPKLIGTILTYLVILYQFQSSEKKP

>DpuGr30

MGLERLNILFFLINATGMLPYRMMLDKKKKRFERFDIGWRHPVTWWFVVVLIIQLIYTPYITSMSVLREISASKNESVIYIMATVLWFVCYGIVKLIPTILLFRVGGFITALTDLKKVDRILDKIPNYQCSSRQRTVIGFVCGIIMIVSMQISASYQTILPVEKTEILLYFFEFFIDFITSLQLTTWLLLIHLTYYNIAHRIAILAHFLGDDQEPIRGGKKITVVPLKAITIEPDTGQLSFRLQKVDYVFDRLAKTCNQLKSVFSFPALLILLTSFLSCTINIFLLLHYFIQPNSAQNNILLYLVLLTSQIAMALVIVVSAGLPAQEVKRLRGKFLRLSTKEIDLNDEAQCAAIVMALSEERVQLSAGGLMPIGKELLPSLTATVITYLVILLQNAIHKKSIQDKGTAVNR

>DpuGr31

MDITLGEISYVANNMSVFEKLGPFVSLCQACGMIPYSIKINSITNQFEKFTFSFKHFNTWWFFFMLVLQLSLFSAAPLFYQEVFQALLSDRTMPLIFTILTGTVSFSYLAQFLTCRWITLRYKQLRNAVEAVQEVERLFGEIFIAQHQSSSLVTRTAIGFTLAVTTSTVALCVMFPIFQSFLPTNLDMFTATAFFSILTSINIMFECQYLLIHLSYYIIAHYIKLLSLHFDAVEDDQLPATLNNRAEKKLEGNALIFAHLCRASSQLNDIFSGPVLFLLTTKFLSIVTCAYAWIFNLIHANEVLDDFSLVFPFLFFTDWIRLLILLSTADMPAHQVRLLRERLTSATHSKFAQLTDSMVITLLQINEDRVRLSAAGLFQVGVHLIPSLIGAVVTYMVILLQN

>DpuGr32

MSVFEQLRPFVSLCQACGMIPFTVEQDLISKKFVRFTFSLRHLTTWWFILLLVLQLTVSVGLIYSSMSQTISLSIDPDLPIMIAVVFGVNWVSISSQFLISRWIVSHYRQLRRAVEAIQEVERLFGDPFIRQHKSSVMTRFIIGFILVVTTATGTISVFTPIFQSVLPAAMGALPIKTAILSFVFLIFVMFDSTFLLAHICYYIIAHYTELLLPYSSFGVVDKDQIPTSSRGMENMLSRSTLIFKYLCRASSEINYIFSMPALFILLTKFLAVISFAFAYIYTFSHTHEVIENYVLALPFLFFVDWIRILILLSAADLPVRVFREHVIAESCSEEVQAIMATLIQIDEDRVRLSAAGLFNVGMFLIPGLTGAAVTYMVILLQS

>DpuGr33

MVTSAGIITNTFKCNHQQINRIVNLQSRSYKSVAKPFLIMSVFEQLHPFVSLFQACGMIPYTIERNLATQKFAKFTFSFKHRTTWWFVLISISQVLCIVAMSFTSNNVGESLSTDKTMPVTVLILFSVTHMSRIAELLLSRCIVLRFHRLRNAVEAVQEVERLFGDKYLAQQNRNSITARFIIGFILVNLPAIGMLFVSISALESLSSLANSNILLITSMMFMLTLIYIMIDCTFFLSYLCYYIIAHYIQLLVIHSDMTEDNELQMNKRDGNFKTLRRSAKIFDTLCRASSEINDIFSAPVLFLLTTKFISVVTHAFIYIYSFIHENAVLESGNIAFPFVFITDWSRILVILASADLPVRLLRERMSAVMSRSRFSKKLTDNVEAMTLLLQIDEDRVRLTAAGLFKVGMHLIPALTGAVVTYMVILLQN

>DpuGr34FIX

MSVFEQIRPFVNLCQACGMIPYAIENNSMTNTFSRFTFSFKHRVTRWFLLILALQIVSIGFASYFAANKPGSTISTDHSLPITIKILSVVNSISSLVQLLFCRWLVMSYRRLQRTIEAVQQVERLFGEEFIAQHKSSLATRIIIGFILILTTTVGGTVVVVPLIIPFSVLENPIVLLGTAAILLIMMMTATMFECIFLFIHICNYIISHYMHMVLLDSEADRNDDHLNWAENLKILRHNAKIFHYLCRASSELNHIFSFPALILLTTKFVAVINAAFIFVYYFVHGNDVLQHYSLVFLFLFFTEWIQILVLLFAADMPVTQVCLVRERLIRMSISRFARTSAEKIAVMSILMQIDEGRVRLSAGGLFNVGVHLIPALTGSVVTYMVILLQN

>DpuGr35FIX

MQIFTKLRLFFGFCQLFGVIPYSVETEEFPTKTKFKKFSFSLRHPVAWWFFFLGTFTAVVSCWDFKMIWNVLHEDDGVLRSHVSRIISVFVLQEHIFYVALMVMSRYVIIKYVCFRRTVYFLQKINSELRVDDMSPDGIPTVTRSAMLSIISAIVGTIALIASNTALFNLKVENEGLIPAVMVVVGRFFISIMLISTFSIIYLCYKITACYIQFIILDMKRPFRLSPTENVPEEKQFAWIQVVDTNAILSWKYKLRHSKRMFGYLCEAITNLNQVFSYPALVFLTLRLVSSAFSLYIIINGLMNNNNEFFQALIPACVANSIVSFLSVFVVLKATESPNVQARHLRERIFTILNEETDLQMDDELEALLFLKNISEERIRLSAAGLFHVGLNLIPSLTASMITYFIILLQTKFD

>DpuGr36

MSVFEHLRPLVSLCQACGMIPFTMEENVISNKFVRFTFSFRHRTTWWFMVMFVLQFVLQFVMVKLSVSILDGLINDENVPMTVTILSGITMFSFTAQILLSRWIALNYRKLRNAVEALQEVERLFGEKFIEQHQCFLGYRFVIAVTLIVTTVVFSFVVMASLFQPFYPADMGLMPTVALYFMLSLVNVMIECTFLLIHMSYYVISHYIQLLFLNSGKSDANGLPVASRKGAENIKELRQNALIFDYLCQASSELNDLFSVPVLFILTVKFVTVVSAAFTYIYSFTYSNLIIENVLFVSPFLFVCDWIRLLVIFTAADMPVNQVRLLRERLCARSYSRFSQTLAENVAEMTILMQMNEDRIQMSAAGVFKVGVHLIPALVGAVVTYMVILLQN

>DpuGr37

MSIFEQLRPFVSLCQACGMMPFTIEPNLITNKFEKFTFSFRHFTTWWFFLVLFVLQPAFFFVMRNMTKDAPTDLLYGQDMPITVHMMLFVSAIGTLVELVLSRWIVLQYRKLRYAIEAVQEVEQLLGKKFISQHQSSVTARFVIGFILAISVSVGCVIANEPAYEELFPKNVNVFLMTFMYFVLVLLFVLFQCTYLLFYMCYYVIAHYVQLLLHPDVKERRIISLNAKESGDVMRRNVLIFDYLCRASQELNGVFSIPILILLTSKFMSVVAFAFAYIYNKFIHYDDMLENHSMMFASMFFVYWIQILVLLTAADMPANQVVRLLRERATAISSSGLSQSLDGKLLAMTSLIQIDEKRVRLSAAGLFEVGVHLIPALAGAVVTYMVILLDT

>DpuGr38

MRVFLQLRPFVWFCQFFGIIPFYMVIDDSPAKTFEKFSFSLRHPVTWWFSFLFISLIVLPVLDIQVVWQIIPHTDDAPTHVPPIFLGFILQEHVFSFILLASARYMICKHACIRRAVSLVQKVNNELMVEDMPSEYIPAVRKHVIAAILSPLIASISVMVSNPTIFNSKVQTAGLAFAVLNVISRCIMIVMVMGSFLLSYLCYYIVSCYIRLIMSNLERLQRLSEVEMLVEMVSETRKTAWIPMSYTRKQKLKNSKRMFGYLCEALVELNQAFSFPVLIFLTLRLISSAFSLYVAISGLLNANNLILRALVPSFATISIIGLFGILIVFRAADLPIIQMRELRERIFSILNEEKDLDMDMELEAVAFLQDISEERVRLSAAGLFSVGMNVVPSLTAAIITYLIILLQAKLV

>DpuGr39

MVTQTQPKWDQQAIHFPHSSFSFTHKSTAKIIAQTKMSVFDQLRPFVSFCQACGMIPFTIEQNQITNKFAKFTFSFRHLTTWWFFLVMFMETVVMSVMGYVSFIQTFYILNGQNLPITVIILLFVIGMSYFAQLLLSRWIILHYRQLRNAIETIQEVEKLFGEKFISQHKSSVTTRFIIGFVLVVISAIGTLTLSTPIHRQFSSLIYTNNVFASVATFSTLAFIYLLFESTFFFFHMCYYIIAYYVQLLIIRSDAEDQTISQKKKENMGVLKENTLILNYLCRASQELNRIFSFPVLILLTAKFISVVTTAFAYIYNCFIHSNVVLDSYSWLFLFTFFTDWIKMLILFNAADMPVNQVIRLLRDKVTAISSYGLSHSLAEKFAFMTSLVQIDENRVRLSAAGLFKVGMHLVPALAGAVVTYMVILLQN

>DpuGr40PSE

MRVFLQLRPFVWFCQFFGIIPFYMVIDDSPAKTFKKFSFSLRHPLTWWFSFLFISLTVLPVLDIQVIWRILYHADDVPTHIGLPPFISFILQEHVFSFILLASARYLICKHACISRAVSLVQKVNNELMVEDMPSEYIPAVRKHVSAAILSPLIASISLMVSNPIIFNSKIQFSGLSFAVLNLISRCFIIVLVMGLILLCYLCYYIVSCYIRLIMSNLERPQRLPEVEMLVKQVSETRKTAWYIGTCFLRIDFLMFLSHFHRCTLTVGWQXLVELNQAFSFPVLIFLTLRLISSAFSLYVAISGILNADNLIFRALVPAFATISIVGIFGILIIFRAADLPIIEMRELREKIFCILTDEKDLDMDMKLEAVKFLQDISEQRVRLSAAGLFSVGMNLVPSLTAAIITYLIILLQAKLV

>DpuGr41

MSVFEQLRPFVSLCQACGMIPFTIEQNQITKKFAKFTFSFRHLTTWWFFLVMVMETVVMGVMGYFSLTQSLDLLMVQNLPITVIILLLGIAISYAIQIILSRWIVLHYRQLGNVVKVIQEVEKLFGEKFISRHPSSVTSRFVIGFILVITSAVCALILSSPVHRQFSSSIYVYISSSTAMFFAIALIYALFECTFFFFYMCYYIIAYYVQLLIIRSDVEAIAVKQTTSRKTKESTGMLKENILILNYLCGASQELNGIFSVPVLVLLTTKFISVVTSAFTYIFNYFIHSNVVLDSYSWWFLFTFFTDWIKILILLTAADMPANQVRLLREKITVISSSEMSQSLAEKIALMTSLVQIDENRVRLSAAGLFKVGMHLVPALAGAVVTYMVILLQN

>DpuGr42

MRQQKDLFSPGLAHYRRYPSPHQILPLRLVFSCIGRRDVNCNSARARIRNESFCNVECVKSNISLDETLRPLWKLTHYCGILLDWCRPISKNNHRFYKVSRFIWITLLFLLLFAIFSFEIIQLVKGIESAPNIHVMIPNILWSVPLLVGFIVQEQFLRYRREFLRFFKDWRALEIEIAQSNPDCVMCQSRGMHLTMYAIHAVMTIAGLIALGLDIFNHPEAPYLISTYQIVRETIPPPLICLIHLTPIGFTLTFLVIGDLVPSFTYYHAALAVNCLEKNMVKFCSKHLSSDRVLFVKSLANKPLINQNLNYFKTLPETSPYELDGYVHLIWKTYDNIDRMVNRANSLFGTFWVCSQGVILFMITSLLYSVFYYLDDALKMRSIDQILPYLLNFIGLTFRLISSTLISSHLNQRTIQFRGTLNRLLNQHWFQLSKPDRELLRSFLSRLSNDHLVASPLDLYNITPSILLSVLGLVVSYVIVLLQSN

>DpuGr43

MANKIQDVEMLSRLRNQRIRKANGLATNITLDETLRPLWKLTHYSGILLDWCFPISNNNPRRICKFLRYFSIALSFSLLVAVTSFQLMQLLLGIEKAPNIHAIIPNMLWFIPIVLGIVIQVHCLRLRRKFLGFFKDWRQLEIEIVHLNPNCIMCKSRKMHLMMYGIYVVITLASVIALGMDISNNPESSYLISTYPAVRQVVPLIVIGSVHLTSIVLTWILTSLGDFIPSFTYYHTALASCLENDVRALFDKGKDADDLFIQSRLSLLAGKLDPLKKSLSTELSTWQLDVPILRIWTRFEKIGKMVSRADFLFGSFMVYSQAGAIFFITALLYSVLYNLGDALRMRSVGPVLSYTLNLLAILFRFFSTMLISSQLHRSVGKFRTALNDLLSQNWNRMNKGDRDLLRSFLWRLQADPLVAHPLGVYKVTPSVLLSVFGVIVSYVIVLLQSK

>DpuGr44

MDNISNVLPFPYLDRIATVKHEENVSRRSVDNQRLYASVKETLRPWWVLTRYCGILPDWCCHPHNIDRKRLDAVAVTLIRYTAVAFVFSFVFSMMIFQLSRTVVAVQSMTTVHSVIPFLLWLSAIPLAVVTQIWYIARRRDLLAFFKDWDLFEKYLDVRYSHQELVYPMVRKPRILVYTSKLVTLLVLVVGLSMIIFNFPDAPYLLTNFQALRDVIPIPILGFIHLVNVILVLGLTTLCETVPALVFFHFQCQVHVLQREFDNVFKLFNSQCNYHKLSGRLDFDPGLFFAAQLRQLFEFYETVRRFVGRANSLFGVLLFLNHGMRLIVICVMLYSVLYLLQSTPVTAGIYLINFCIHLCELVTSTLLTAQLYRASDRLRTHLAVLLTRYWDSIPKEERKLLVHFNGRIQTDPLAATPLGLYNVTPSFLLTVASLAVTYVIILLQSK

>DpuGr45

MVREKANHIEIGRTRGLTLKDTLHPWWVLTRYFSIMPDWCHPYHDRDRPVLNAFRCIGIAVVMGFISLMLIFHLTKMVMAILVMTTLHSIIPYMLWLSAIPLALFTQLWYVYYRREFLDFFRDWGHFEKELMLGYNHHDMFSTIKKTRALIYTSRLLMLIGLLVGMGFVVFRKPDVPYLLSNLQFLRQIIPVPILCSIHLVNITCVVILASFCDSVSALAFFHIALEVRVLLNEFERIFTILDPLNHDEKMTDQLELNRGYLFATHLRQLFDYYETLRSFVTRSNSLFGVLWFLNHCMRLSIICIMLYSVLYMFQSSPEDAGIYLANLLTNVYELVICTLLTTKIYCASDRLRSVLTVLLTKYWDLIPNGERELLIVFIGRLQTDPLAASPLGLYNVTPSFLLTITSLTITYVVILLQSK

>DpuGr46

MVHSKPNIPVYQDVMFLFRFISFTGASTFVPYGEGMQNPRISTVLVRILQQITCLAIFLLLLAMSVFEFIQFVTVIVKMKNIGEVIPNIIWITSFPLAMGAQAFYIFQRPKLLKFFENWQDIHTMYSESNGHVTERQTRPRMVCTSVAMVIVLILDFPILPFLPSTYEPLAAIPLHTNNLYLTCALFGVSLFYAWNLSAASDLVPSMIYFHLALSLKKTSEFLKEPQNVLDSQTIENNCKNLEVDETNFNSHPSAVKGSQDSNQVIWQKGWLYYEAIRKLINESNSRFGSMLLLNHGAMFFVASSNVFSILAWYRGMSWLLLLVNGMNAVATILRFITTILLCSRLYAAGEHLQSNICRFLAVTWFRLKPDERQFFTAFLVRVQDDGIVASPLGLYPIRPSILLALLSLMVTYLIVLLQSSEVSTFSEYGNFTGLITGTRFYNPLNV

>DpuGr47

MVAETLRDRFLRLALSPLSALLKIVGVFYNQNLDSQQTQRLCRSGTFFLLILAIQSNIYIFVRRSQIIEILFGSQEINVDKLIEVLVHELTRFSQLVSDAIVHLSLVFKIWPSVTLFLETLESVDSQFGQPSLSPIKRYSLFGLIYMLFMVVLNWILFTHFDLNHPVRLAYWLDMLQNSIKIFSNLCLTVLPVWLFIICGQLYIFYTRKLIANLKLLYLMVRNATVPSGTAVQRTFSNLRSLKHLYLAAELLHRHFSTVLAVNCFLTFVTMFTSSYYIIEFYKRGNVIVVCWSCIDVLEAFLRFWLICHTSDQIREITIECITVLRSLRDTRNEGNMSECNKITSYIIEISQMSKNLDRHSLNGVLSLSKSLIIPTLEIIFTYLLIIYQFKSAGEGTS

>DpuGr48

MAGTLKDRFLHSALSPLNILLQITGVFVNQNSEAQWRRRLYRFWTFVVLVLAIQSNFYVFVKRTLIIDFFFGSQEINVDRFIQVLVSELIRLSSLVSDIIVHLSLVFKIWPSVTLFLETLDSVDLDFGRPNLSPIKRYSIYGLIYVLFTVVLNCAFSIYWDSTHPNKPAFWLEMFQNLIRLFSNQGFTVLPVWIFIICGQLCIFYTRELIANLNLFCAMVRNAGVIPSRIVAQRTFSNLRSLKQLYEAADLLHRHFSTILVVNCFLTVVTMFTSFYYLIELFERGYVVGACWEVSDVLDSFVRFWLICHTSDRIRETSTECITVLRSLRDNRNATTLSECNKITSYIIEISQMSKNLERQNLKGILSSSKRLIIPTLETIFMYLLIIYQFQSAVNCQLSDLYQCVT

>DpuGr49

MAGTLRDRFLLSALSPLNILLQITGVFVNQNSETRWRQRFYRFWTFFLLILAVQSNIYIFVRRSFMMEQIFTYQQINVDRFIHFLVNALIRLSWAVSETIVHLILVFKMWPSVILYLETLETVDFNFGRPNLSPTKRYSLFGLIYVLLTVALNWILSIYWDLNHPVRLVYWLDVLQNSIRILSNHCFTVFPVWIFIICSQLHIFYTRKLIANSKLLNFVVRNATVPISQTDVAQRTFSNLRSLKQLYLAADLLHRQFSTILMVNCFLAFITMLTSSYYVIEFSQLGYVVIVCWEASHVVDSFVRFWLICHTSDRIRESTTECISVLRSLRDTRNAEKLSECNKITSYIIEISQMSKNLERHSLKGVLSVSKRLIIPTLEIIFTYLLIIYQFKSAGNK

>DpuGr50

MAGTLKDRFLLSVLSPLSILLKITGVFVIQNAETQWRQSLYRFWTFVVLISAVQSNIYIFVRRTLIIEFLLSGQQINVNRLIQVLLNELIRLSALVSDTIIHLSLVFKIWPSVTLFLETLDSVDLDFGRPNLSPIKRYSIYGLIYVLVTVVLNWIFSIYWDLNHPVRLVYWLDMLQNFIRIFANQGFTVIPVWIFIICGQLCIFYTRKLIANLKLLNFVVRNATGVPSRIVAQQRTFAHLRSLKQLYEAADLLHRHFSTILAVNCFLTVVTMLTSSYYVIEFFKSGRLIVLCWDASDVLDSFVRFWLICHTSDRIRETTTECISVLRSLRDAGNEGNLSECNNKITSYIIEISQIRKNIERHSLKGVLSLSKRLIIPTLEIIFTYLLIIYQFQSAGEGSP

>DpuGr51

MAGTLRDRFLLSALSPLNILLQITGVFVNQNSEAQWRQRLYRFWTFFLLILAVQSNIYIFVRRTLIIQLLPTIRQINVDRLIQVLLNSLIRLNGLVFDTMIHLILVFQLWPSVILYLETLETVDLDFGRPNLSPIKRFSLFSLIYVLLTVVLQWIISTYSEFYHLNRQAYWLDVFQKVIRVFANQCFTMIPVWIFIICSQLHTFYTRKLIAKLKLLCVVVRNTGVISSRTAVAQRTFSNLRSLKQLYLASDLLHRHFSTILMANCFQTFITMLTSSYYVIESFRKGYWVYFCLDGSDVLDAFVRFWLICHTSDRIRETITECIDVLRNLRDTRKAVDFRDCNKITSYIIEISQMSNNLERHSLKGVLSLSKRLIIPTLEIIFTYLLIIYQFQTK

>DpuGr52

MVLLFKTRFLQSTRPFSFYLKTIGVFLHLTNKPVQRFSCLIFSCFSFALTFQAGLYVFVERSVLDIRYAFSGESGRGINFTKQFSRIDAFATGLATTNPFVCGSATSLLLVVTSRETVRLFCFSMGTMDRLSKRPDLKSLWRYSIAAVTWIAITCGGSIYLMLLNPDWVSQNLLMCFQSLIILTQSLNYTLSATWIYTLCARTIVIYYLRIADELQRLARKDCDYCLNNEMVRILNMFKHLSQATRFLHNRLELILLINCYYLVANLIYLMYYATCFTSWKTFIIDWFSLVEGICRLFLICHSADSIRSSRIQSISILRQLRDDSVFTTASADRTKLTLFIIELYNCEKDVTLLGMMQLSKRIILKAVETALTFYIIIYQFQIGWSQLPAE

>DpuGr53

MAMNKMSKEAWAAKPSLGYETGDLETILGPVFTLFRCFGINFHEPSDTNCRFLHQTAQIGWVVLTLGTCGFRLLVYFPSILYRSIDDVENSKTVKSIMHYLIEFLNYWIYILAVFIGSWCMARWSAAKLWRAIEDMLLHIPLSPEDTARCRKTSVQCIFGCLVALSLNLILSLSIEISLAAPANRMALALLSVVYFYSFGSCALFISAGRIISIAFQTVSSQLELNCCPPSTNAAVVWNYQRQFGIACRAAEKFCSTFGFVLFATVSYTFIGFVNASYNLLKSYQNLPQGSDGGEDLYYIIPRTIRLTYMIVEHLFRLWLICHTADLIRSKALSLVPVLQSIRNDLYSRQGCNDESQEVHTFLLEVSETSPTFGVLGMFTLTKHIIISLIGTTLTYLVVLCQFSTADNPVAMTPISNASQSS

>DpuGr54

MASFSEAVEPILKFLQFCGIHFQLDDELVSGRRGTRYFLRFYAAVCLTLNIGWTGYCYVKYAPILTLSTMNEFVDYINFFLGNVLAHAVLYSMTFGSFAEIISAIERLERLLEHDAHYYRKLRRMSFIAVTLIVAFVMAQIAMTLYRQKWNWLKTSSSISGELCMFYPMASYCLFFISALSLAGSFQHVNSRLAGYLQGGSIKAANNLDLADIRANHGLVCQAVDQLNGRFQALSLIHVVFAFTGFVNTSFMLLMGEYTFLKVVLYQLECVARLALLGYACDRIQSEALACLKTLASVRSDHITIHSLLHQQQQLMLGGQVLQTAPTFDVYGLFHLKRQLLPKLIGTTITYLVILYQFKGTPT

>DpuGr55

MAKKPDVIQLWNPVISFLRCYGVVPLKQCDKDPYFERSSGSFCWCLSVASIYLFTFAISVCLVIDTFNSSSKMIADATFYLIYYAHCEMTLVFFLYYSGELVSLFQHWVETERQLQLRKIFLGKTTKAQCWLIFIATVILSNLENGFYITGAVMDAKNMTEIIYRLVNLAGKGTDLSPYFGGYKDVYAFALIFVESLSEVAWIAGDLIIALISILLRRYYEALNRQLRSQHYAGVISLRQLEEIRRVQLAISTLVQKIAEVFSPLILITIGCNVVYILTFLYSGLEADITSPSFFVRFVFTYSFIYIALRLTFSVYLASLLNEMPRNTIHYLCGLPSIVGNNTEQQRRRLIKMDLIIEEIQSESTALGGGGFFILSKSSAASLFSLIVTYEVVMLQLPHK

>DpuGr56

MKKKPDFIQLWNPVISFCRIYAVIPLRQSDKEPYFERCSGSLCWCLLVGSIYLFGFVLSVVLVLDTFSSSSKIIANATFYLIYYVHCEMTLVFFIYRSQDLLDLFQNWIQVENLLIKHKIHLGKVLVAQCWTIVIATLIMSHLENGCYIISAVIDAESLSDTIHLFVNLAGKGTDLNPYFGDYKDIYGFFLIFVESLSEVAWISGDLIIALISIICRRYYEALQEKLLSDNHHSFRQLEELRQLQLAISTLVHKVTEVFSPLILISVGCNVVYILAFLYSGLEADLFSPSFLVRLIFTYSFIYVVLRLIFSVFLASRLSEMPEKTIDYLYSLPSIVGSNSDEQINRAIKMIMIVQEIQSNPISLGGSGFFVLCKSSVAALFSLIVTYEVVMLQLPR

>DpuGr57

MGGSQRSTVTLRALIPYLVIYRALGLLPMNFAPETANNPSKPTTPWLCRFRPRLCWRQIGWTRLYSASVFLLVTGFLFEKLPSLESKGKDVVGRLEVANRSSICLNVFVTSAICFCYVGKRMSALIQKMIQCEHDLIALGCHLQNRLAVYCWMVLAIGSVATILNELVIGIRYIRPPMNSAIEEEDQSTLLVIFRSIMTPFTAGLMPFVDFSVIHLSLIFSAYLKQLRKKIEHWSSLGLKAKERLRLTYWKVQSLIKEADDILSPTILIESLSCVAHLTLQINILVGTKIEPSESDEYDKHHVLMVTAITTVYLFIRFISSSLFAEMIHRERFKSQRCLYEQSGEEFWSYDLELLVTKRFVHQLTLPLGFTGCGFFKLGKSYLIAMCGTILTYEIVLMDMANSENPATFESCVNNSFTLAQHGTGIAENSN

>DpuGr58

MASGELFRSLSPLFWVLRVTGGTPFVHSDSFAGMLGFQWCHPQTFWFAFVTLVHVSFISAQVFGGLFSLGDKSDNEIDEPGAHFVTNPTAQLTNSISQARFLVDTFVLSKFIHFQLASFQIFFEQMDSVDRAVLSVVTRRTRKMIIFAISIYLFWDMIFLIYGFDGIFREHGKDSAWMFAGRITINLMHIIGQLHSNMLVILFASLCYIIGLRFAGLKEILQQTVVIDEGIFRHLWNNNFDSHNASPSITQPSMQNRVLPDNESLPDVGPPITILDGLRLRPLSSRLVFLKTVYLSLSEAVLVHFNQVFGLSLLFYLISKLISVSIGCHSFIHHIMKNLNIGLFWCLTNIPDITAMFIIFQSADYVRNQVAGTIATYILVLFQFNASEDVINADVKGSVI
